# Psychological and Cognitive Markers of Behavioral Variant Frontotemporal Dementia–A Clinical Neuropsychologist's View on Diagnostic Criteria and Beyond

**DOI:** 10.3389/fneur.2019.00594

**Published:** 2019-06-07

**Authors:** Andreas Johnen, Maxime Bertoux

**Affiliations:** ^1^Section for Neuropsychology, Department of Neurology, University Hospital Münster, Münster, Germany; ^2^Univ Lille, Inserm UMR 1171 Degenerative and Vascular Cognitive Disorders, CHU Lille, Lille, France

**Keywords:** bvFTD, cognition, diagnosis, apraxia, social cognition, neuropsychological assessment, interoception, behavior

## Abstract

Behavioral variant frontotemporal dementia (bvFTD) is the second leading cognitive disorder caused by neurodegeneration in patients under 65 years of age. Characterized by frontal, insular, and/or temporal brain atrophy, patients present with heterogeneous constellations of behavioral and psychological symptoms among which progressive changes in social conduct, lack of empathy, apathy, disinhibited behaviors, and cognitive impairments are frequently observed. Since the histopathology of the disease is heterogeneous and identified genetic mutations only account for ~30% of cases, there are no reliable biomarkers for the diagnosis of bvFTD available in clinical routine as yet. Early detection of bvFTD thus relies on correct application of clinical diagnostic criteria. Their evaluation however, requires expertise and in-depth assessments of cognitive functions, history taking, clinical observations as well as caregiver reports on behavioral and psychological symptoms and their respective changes. With this review, we aim for a critical appraisal of common methods to access the behavioral and psychological symptoms as well as the cognitive alterations presented in the diagnostic criteria for bvFTD. We highlight both, practical difficulties as well as current controversies regarding an overlap of symptoms and particularly cognitive impairments with other neurodegenerative and primary psychiatric diseases. We then review more recent developments and evidence on cognitive, behavioral and psychological symptoms of bvFTD beyond the diagnostic criteria which may prospectively enhance the early detection and differential diagnosis in clinical routine. In particular, evidence on specific impairments in social and emotional processing, praxis abilities as well as interoceptive processing in bvFTD is summarized and potential links with behavior and classic cognitive domains are discussed. We finally outline both, future opportunities and major challenges with regard to the role of clinical neuropsychology in detecting bvFTD and related neurocognitive disorders.

## Introduction

Behavioral variant frontotemporal dementia (bvFTD) is a neurodegenerative disease characterized by early progressive changes in behavior, social conduct, emotional processing as well as specific cognitive impairments (1, 2). Accounting for this symptomatology is a pronounced and relatively focused neural loss in bilateral frontal, insular, and/or anterior temporal cortices that can typically be found in patients early in the disease.

bvFTD is the most frequent clinical syndrome of FTD (which also includes two other clinical dementia syndromes with predominant language dysfunctions). Fronto-Temporal Lobar Degeneration (FTLD) is the broader pathological disease spectrum also encompassing FTD, motoneuron disease (MND), progressive supranuclear palsy (PSP), and corticobasal degeneration (CBD). Estimated prevalence rates highly vary depending on the employed diagnostic criteria which have been considerably refined during the last two decades (1–3). Prevalence rates are further clouded because bvFTD, FTD, or FTLD patients are not systematically considered separately across studies. The average disease onset of bvFTD is estimated within the early sixth decade of life although patients can also be substantially younger or older as some cases with a disease onset in the 20s and after 85 years of age have been described (4–6). Within the most relevant age range of 45 to 65 years, 10–30 in 100,000 people are estimated to be affected by FTLD (4, 7). Converging evidence then suggests that bvFTD is the second most frequent young-onset (<65 years old) cognitive neurodegenerative disorder following Alzheimer's disease (AD) (4, 7, 8). Due to relatively large symptomatic overlap and the lack of established biomarkers, the prevalence of bvFTD may well be underestimated because of frequent misdiagnoses with either a different neurodegenerative cognitive disorder or a primary psychiatric disorder (9–11). Half of bvFTD patients indeed received a prior primary-psychiatric diagnosis (9, 12).

As FTLD is a heterogenous pathophysiological entity, bvFTD can be caused by different underlying pathologies (13–15). Most of the cases include the abnormal intraneuronal depositions of aberrant forms of specific proteins such as tau (FTLD-tau) and TAR-DNA binding protein 43 (TDP-43; FTLD-TDP) in equal proportion, and less frequently, fused-in-sarcoma (FUS; FTLD-FUS) (16, 17). Currently, no methods allow the detection or prediction of FTLD pathologies during the life of patients and no reliable biomarker exists for the diagnosis of bvFTD, although some associations between genetics and post-mortem histopathological findings have been established (13, 18). While the majority of cases with FTLD are estimated to have a sporadic form, a positive familial history may be found in up to 40% of cases (19, 20), with a suspected autosomal dominant pattern of inheritance found in at least 10% of patients (21, 22). Although other rare genetic variants have been found, mutations in either the MAPT, GRN, or C9orf72 genes account for the vast majority of genetic variants of FTLD. Interestingly, the hexamino acid expansion on the C9orf72 gene is also very frequently associated with families presenting with MND or a combination of FTD and MND (23, 24), suggesting a continuum between both syndromes. Overall, this underlines the importance of a detailed family history during the clinical interview.

On the anatomical level, meta-analyses of the atrophy profile of patients with bvFTD found significant clusters of reduced brain volume in frontomedian areas, in the superior frontal sulcus, parts of the thalamus and insula as well as in striatal regions (25–27). Focal frontal atrophy is a positive marker of bvFTD but can nevertheless be absent or very subtle during the earliest stages of the disease. In those cases, 18-Fluorodeoxyglucose-Positron Emission Tomography (FDG-PET) has shown to enhance diagnostic certainty, by evidencing a regional hypometabolism in similar frontal and anterior temporal brain areas (28, 29). Multimodal imaging approaches simultaneously combining hypometabolism and brain volume loss also showed promising results while being usually not available in clinical routine (30). Interestingly, differential patterns of early brain atrophy have been observed in the genetic forms of bvFTD as well as in asymptomatic mutation carriers, sometimes up to 15 years before the onset of clinical symptoms (31, 32). An atrophy of the hippocampus and amygdala may be observed 15 years before the onset of the disease in asymptomatic MAPT carriers for example (32).

### Aims of This Review

As a result of the heterogeneity regarding the underlying pathology of bvFTD, there are currently no reliable, specific, and established biomarkers available to clinicians. A correct and early diagnosis is nevertheless crucial for disease management, initiation of treatment as well as support of the usually highly distressed caregivers (33). Differential diagnosis strongly relies on the correct application of the current FTD Consortium (FTDC) clinical criteria and exclusion of other causes for the symptoms. Recently, however, critics have been raised toward some parts of these criteria, particularly toward their ability to guide the differential diagnosis with AD or primary psychiatric disorders (34, 35). Here, we thus aim for a critical appraisal of the current clinical criteria for the diagnosis of bvFTD. We will highlight strengths, practical difficulties as well as controversies of these criteria, give real-world examples and provide suggestions for their practical evaluation in clinical settings. We then aim to review more recent developments regarding early clinical detection of bvFTD beyond the established diagnostic criteria with an emphasis on in-depth neuropsychological assessment of specific cognitive domains (i.e., social and affective cognition and praxis abilities), physiological alterations (i.e., interoception) and potential links with “classic” behavioral and cognitive symptoms of bvFTD. Finally, we outline current challenges and opportunities for the field of clinical neuropsychology regarding its crucial tasks to develop and establish novel and clinically meaningful “cognitive markers” in order to use them for (i) staging of disease severity, (ii) validating future biomarkers, and (iii) identifying the precise cognitive dysfunctions that will need to be remediated in future disease-modifying strategies for bvFTD.

## The Clinical Diagnostic Criteria for bvFTD

After an initial paper in 1994, the first comprehensive consensus criteria for bvFTD from the FTDC, an international group of experts on FTD, were released in 1998 (2). These criteria enabled clinicians and researchers to focus on a set of four key criteria for clinical diagnosis, namely a decline in social interpersonal conduct, impairment in regulation of personal conduct, emotional blunting, and loss of insight. Each of these criteria had been standardized by a brief text definition. Key symptoms and definitions have since been consecutively revised and refined and the current set of criteria (1) now reaches high sensitivities and specificities when tested against competing neurodegenerative diseases (by post-mortem pathological confirmation of either of the known underlying FTLD pathologies) as well as good inter-rater reliability (36, 37).

Despite consensus text definitions of the FTDC clinical criteria, clinicians today still face the problem that the criteria have not been standardized by means of “tests” or clinical scales and even the methods of their assessment (e.g., patient anamnesis, clinical judgement, clinical observations, caregiver anamnesis, standardized questionnaires, cognitive performance tests) are not consistently specified and likely differ considerably across centers. In addition, some studies have underlined a substantial overlap between symptoms of specific primary psychiatric diseases (e.g., late-onset unipolar or bipolar affective disorders, schizo-affective disorders) and symptoms of bvFTD. These shared symptoms may explain why bvFTD patients are frequently misdiagnosed with psychiatric disorders as outlined before (9, 12), but they also explain why, on the basis of the FTDC criteria, a significant proportion of psychiatric patients could be misdiagnosed as possible bvFTD (10, 11, 38, 39). Related to the issue of clinical misdiagnoses based on the FTDC criteria and further complicating the picture, a condition mimicking bvFTD has been been described and labeled “bvFTD phenocopy syndrome,” implying that patients may display the typical behavioral symptoms of bvFTD but show no progression and no evidence of atrophy or hypometabolism [for a recent systematic review on features of individuals with phenocopy bvFTD, see (40)].

Another important criticism toward the current FTDC criteria for bvFTD is that some exclusion criteria (e.g., the proposed cognitive profile of major executive dysfunction and spared memory functions) seem overly restrictive and may lead to false exclusions (34). In the following, we will first provide an overview on the different sources of information available to clinicians and their respective importance in the process of diagnosing bvFTD. We will then give practical suggestions to assess the proposed behavioral and psychological (section The Clinical Diagnostic Criteria for bvFTD) as well as cognitive (section Cognitive Dysfunctions in bvFTD) criteria (see also Table 1).

**Table 1:** Clinical, behavioral, psychological, and cognitive diagnostic domains for bvFTD.

**Domain**	**Suggested method of assessment (scale/test)**	**Typical results/observation in early bvFTD**
Anamnesis/clinical and neurological examination		
Motor-disinhibition	Saccade and anti-saccade test	Fails to perform anti-saccades as compared to saccades.
Speech and language	Clinical observation	Monotonous speech with little tonal modulation, stereotypic utterances, use of “foul” language.
Emotional blunting	Clinical observation	Seems indifferent and without adequate affect toward diagnostic process or toward the suffering of caregivers.
Anosognosia/Lack of insight	Clinical observation and confrontation with deficits	Spontaneously reports little complaints or only in a stereotypic, shallow way. Reacts indifferent toward cognitive deficits or caregiver reports of behavioral changes.
Behavioral and psychological symptoms		
Behavioral disinhibition	Clinical observation, standardized multiple-domain caregiver interview or questionnaire (e.g., FBI, FrSBE)	High total appearance of disinhibited behaviors compared to healthy subjects and compared to premorbid levels. Possible inappropriate comments or actions during the examination, favored by a lack of rigidity in the clinical setting.
Impulsive, disinhibited eating	Caregiver interview, standardized caregiver questionnaire (e.g., APEHQ)	Shows a disinhibited eating pattern and prefers sweets and candy (“sweet tooth”), sometimes increased, impulsive consumption of alcohol or cigarettes.
Apathy	Clinical observation, standardized caregiver questionnaire (e.g., ACL)	High levels of apathy and inertia, few interests, usually little suffering or complaints about affective/mood disturbances. Sometimes there is a need of frequent incentives from the clinicians during non-directive examination and a possible over compliance to the clinician's instructions.
Loss of empathy	Caregiver interview or standardized caregiver questionnaire (e.g., IRI)	Lacks empathy for others, seems irresponsible for others; significant changes in caring compared to premorbid behaviors.
Change of humor or music preference	Clinical observation, caregiver interview	Prefers simple humor (e.g., “slapstick”) and less complex types of music as compared to former preferences; sometimes excessive and repetitive consumption of television programs or music.
Rituals and obsessive-compulsive behaviors	Clinical observation, caregiver interview	Excessive collecting or hoarding, sometimes accompanied by deficits in self-care and hygiene (Diogenes syndrome).
Cognitive performance		
Executive Functions	Standardized neuropsychological tests	Subnormal performance specifically in tasks for behavioral inhibition (e.g., Hayling-task) and cognitive flexibility (e.g., letter fluency). Multiple subtest screenings (e.g., FAB, INECO, FRONTIERS) cannot reliably discriminate bvFTD from other neurodegenerative disorders.
Episodic (verbal) memory	Standardized neuropsychological tests (e.g., FCSRT, RAVLT, CVLT-II)	Often subnormal learning curve and deficient free recall compared to healthy controls/normative data. Possible total (cued) recall deficit as well as (free and cued) delayed recall decrease.
Visuospatial processing and navigation	Standardized neuropsychological tests (e.g., RCFT)	Visuoconstruction and drawing usually spared but may nevertheless appear deficient due to planning deficits and visual organization deficits. Use of qualitative strategy scores are recommended
Social Cognition	Standardized neuropsychological tests (e.g., Mini-SEA)	Reduced ability to recognize facial emotions, particularly those with negative valence. Reduced ability to detect and analyse social norms violations and to infer other's intention and feelings (Theory-of-mind abilities).
Reward processing	Standardized neuropsychological tests	Deficit in reward valuation, reinforcement learning, reversal-learning, decreased sensitivity to losses or delayed reward, diminution of normal cognitive/affective bias.
Apraxia	Standardized neuropsychological tests (e.g., DATE)	Deficits in posture/gesture imitation and pantomime of object-use compared to healthy subjects. Relatively more deficits in “buccofacial” praxis tasks (particularly face imitation) as compared to limb imitation.
Staging of disease severity and progression	Standardized interviews with patient and caregiver (e.g., FTLD-CDR, FRS)	Scales are sensitive for clinical disease progression and may guide clinical observations.

*bvFTD, behavioral variant frontotemporal dementia; FBI, Frontal Behavioral Inventory; FrSBe, Frontal Systems Behavioral scale; ACL, apathy checklist; IRI, Interpersonal Reactivity Index; FCSRT, free and cued selective reminding test; RAVLT, Rey auditory Verbal Learning test; CVLT-II, California Verbal Learning Test second edition; RCFT, Rey Complex Figure test; Mini-SEA, Mini Social Emotional Assessment; DATE, Dementia Apraxia Test; FTLD-CDR, Clinical Dementia Rating scale for Frontotemporal Lobar Degeneration; FRS, Frontotemporal rating scale*.

### Sources of Information to Evaluate the FTDC Criteria in a Clinical Setting

#### Clinical-Neurological Examination

A standardized clinical-neurological examination is critical when an initial suspicion of bvFTD is raised, mainly to exclude alternative causes for the symptoms. For example, ~10% of patients with FTD develop MND and 10–15% of patients with MND also meet criteria for bvFTD (41, 42). Because bvFTD symptomatology may also overlap with CBD and PSP, a standardized neurological assessment of (extrapyramidal) motor functions is particularly important. In most cases of early stage bvFTD, the neurological examination reveals few obvious abnormalities. In particular, reflexes and motor functions are usually not pathological, although primitive “frontal” reflexes (utilization, gripping reflexes) may sometimes be observed (43). Although originally described as a classic sign for PSP, a positive applause sign may also be observed in bvFTD and may be correlated to overall disease severity (44). Another frequently described cognitive-neurological sign of patients with bvFTD is difficulty or failure to perform anti-saccades, most likely reflecting an inability to inhibit the overlearned motor response of a saccade toward a stimulus as compared to its opposing direction (anti-saccade) (45–47). Other clinical signs frequently seen in patients with AD like the “head turning sign,” are not particularly indicative of bvFTD (48).

#### Patient Anamnesis and Clinical Observations of Patients

Patients with bvFTD usually have a diminished or absent sense for the behavioral, cognitive, or psychological changes that are reported by their relatives or co-workers (sometimes referred to as anosognosia). Unlike the lead symptoms of other early neurocognitive disorders, cognitive, or behavioral symptoms are thus rarely spontaneously reported by patients with bvFTD. If they are, reports are usually brief or shallow, in a stereotypical manner and without showing a coherent sense of either suffering from the symptoms or being overly worried about them. During anamnesis, patients with early bvFTD may even remain to be affectively indifferent when directly confronted with reports of negative consequences of their pathological behaviors (e.g., on their family well-being) or with negative feedback (e.g., regarding their poor cognitive test performances). Although an anosognosia or lack of insight into cognitive and behavioral symptoms may sometimes also be observed in patients with AD, a striking difference in patients with bvFTD is a frequent lack of appropriate emotional participation or affective involvement during the clinical assessment and diagnostic procedure (sometimes specifically referred to as anosodiaphoria or in a wider sense as a sign of “emotional blunting”). Such typical clinical observations during anamnesis may be used to develop an initial suspicion of bvFTD but are highly subjective and require clinical expertise. Only few attempts on the other hand have been published to standardize behavioral observations of patients with bvFTD in clinical contexts (49) or in home settings (50). Preliminary validation studies of these scales have shown that standardized observations of behavior are a valid method to delineate patients with bvFTD from other diagnoses, however their application require a rigid and time-consuming assessment that is often not feasible in clinical contexts.

#### Caregiver Interviews and Standardized Questionnaires

Caregiver reports are so far the main source of information for clinicians to detect and diagnose bvFTD in early disease stages. As psychological and behavioral changes are the lead clinical symptoms of patients with bvFTD they should be assessed extensively through caregiver interviewing, as these information principally allow for a diagnostic differentiation against other common neurodegenerative dementia syndromes like AD (51). As pointed out however, patients with bvFTD frequently present with recent diagnoses of depression, bipolar, schizo-affective disorders, or “burn out” syndromes due to an overlap of similar symptoms e.g., reduced activity, apathy, loss of interest, and in some cases euphoria, agitation, and restlessness (52). To enhance diagnostic accuracy for early bvFTD, it is thus particularly crucial to delineate suspicious behavior and psychological alterations from premorbid personality traits or episodic primary psychiatric disorders. Psychological and behavioral symptoms need to be clearly identified as novel, qualitatively different compared to previous behavior and most important, progressively increasing.

Standardized clinical rating scales that aim at staging symptoms severity in patients with FTLD may thus help clinicians to guide and structure the caregiver anamnesis as well as quantify longitudinal changes of symptoms. The well-known Clinical Dementia Rating scale (CDR) is available in a modified version in order to include behavioral and language dysfunctions geared toward patients with FTD (53). The scale and associated structured interview is available in several languages and has proven successful in staging disease progression in bvFTD. An alternative measurement that focuses more on specific symptoms of bvFTD is the semi-standardized interview Frontotemporal dementia Rating Scale (FRS), which allows a fine-grained clinical severity staging (54).

Several other standardized scales to specifically assess behavioral changes in patients with frontal lobe syndromes are also available (see e.g., Table 1). These scales differ in terms of administration (e.g., interview, standardized, or semi-standardized questionnaires) and the included sets or domains of behavioral symptoms. In general, standardized scales on behavioral symptoms that focus on the core features of patients with “frontal lobe syndromes” (e.g., behavioral disinhibition, apathy, irritability, emotional blunting etc.) have been found to be more sensitive for the early detection of bvFTD (55) than more general scales that are geared more toward other features (e.g., hallucinations, delusions) commonly seen in specific primary psychiatric disorders [e.g., the Neuropsychiatric Inventory; NPI (56)]. Psychotic symptoms like delusions and (auditory or visual) hallucinations are rarely seen or reported in patients with early bvFTD although particularly bodily delusions (e.g., altered sense of bodily perceptions) might be more frequent in patients that later develop MND and/or have a C9orf72 mutation (57–59). Among the recommendable caregiver scales on specific frontal behavioral changes are the Frontal Behavioral Inventory [FBI (60), also available in a shortened, modified version that may be used as a questionnaire (61)] and the Frontal Systems Behavioral scale [FrSBe (62), similarly available in a shortened, modified questionnaire version (63)]. The DAPHNE scale, specifically adapted from the current FTDC diagnostic criteria for behavioral and psychological symptoms has also demonstrated good psychometric properties (64). Of note, extensive normative data stratified for age and education are usually not provided for any of these scales and translations into languages other than their original ones are rarely available or unofficial. In addition, only a few studies have been conducted to compare caregiver assessments to patients' self-reports or clinicians' evaluations and it is likely that high levels of caregiver distress impact the reporting of symptoms. In that perspective, more education and caregiver interventions are needed in order to both, alleviate the stress/burden and to reduce bias regarding caregiver reports of symptom severity, especially in longitudinal assessments (65). Finally, it seems important to highlight that behavioral symptoms, or the way patients' relatives react to them, may be subject to large variations due to distinct cultural differences (66). Currently however, the field lacks studies that have assessed how such variations may impact behavior in bvFTD or its inception by caregivers.

### Behavioral and Psychological Symptoms of the Current FTDC Criteria

Regarding behavioral and psychological symptoms of bvFTD, the FTDC criteria present five core domains of symptoms, which we will briefly summarize in the following, before we will turn to the currently more heavily discussed “neuropsychology item” of the criteria (in section Cognitive Dysfunctions in bvFTD). Importantly, for a new clinical diagnosis of “possible bvFTD,” it is sufficient that any three of the following criteria (e.g., two of the five behavioral/psychological criteria plus the cognition item) are fulfilled (i.e., a functional decline in this domain compared to former level of functioning is seen). Moreover, the criteria require that these symptoms are new and develop “early” i.e., within the first three years of the suspected disease onset. For a higher diagnostic level of evidence (“probable bvFTD”), the criteria require focal atrophy on MRI and/or hypometabolism on PET in frontal or anterior temporal cortices or alternatively, a documented decline of behavioral or cognitive symptoms over time (1).

#### Behavioral Disinhibition

Socially inappropriate behaviors like inappropriate familiarity or a lack of distance (e.g., staring at or touching the clinician, making inappropriate comments or jokes, being inappropriately jovial) during the anamnesis or during the cognitive assessment (e.g., walking out of the room, smiling, shrugging of shoulders) are often subtle signs of early behavioral disinhibition in bvFTD. Difficulties to inhibit irrelevant stimuli from the environment is often considered as a key feature of bvFTD and patients often struggle to focus on a task because of distractors [e.g., (67)], a symptom that has been linked to “environmental dependency” (68) and to increased stimulus-bound thought and behavior (69). Pathological gambling and personal neglect or reduced self-care (Diogenes syndrome) have been well-described (70, 71), as well as utilization or imitation behaviors (43, 72). Anecdotal evidence also suggests that patients with bvFTD may have early changes regarding their preferred type of humor that may be viewed as a “loss of manners” by others (i.e., patients tend to make crass jokes or prefer “slapstick” humor) (73). In some cases, patients present with more obvious dissocial behavior (e.g., sexists comments, overt aggression) or criminal actions even in early disease stages (74, 75). Law violations as a potential correlate of increased behavioral disinhibition are indeed frequently observed in bvFTD, as indicated by studies conducted in USA, Japan, and Europe. Patients with bvFTD for example commit law violation up to five times more often than AD patients. These could manifest as theft, traffic violations, physical violence, sexual harassment, trespassing, and public urination, thus reflecting mainly disruptive, impulsive actions (74–76). Patients may also exhibit changes in sexual behavior, but usually show diminished sexual drive, intimacy, and display of affection whereas only rarely hypersexual or inappropriate/disinhibited sexual behaviors are reported (77–79). Importantly disruptive symptoms subsumed under early behavioral disinhibition are major predictors for caregiver distress in bvFTD (80).

#### Apathy and Inertia

As a behavioral syndrome, apathy could manifest itself as a range of concepts, such as emotional blunting, poor initiation or persistence, indifference to choices, reduced curiosity, lack of interest, and activities, difficulties in implementing actions and social withdrawal. Diminished goal-directed behaviors and intellectual activities (such as reading) as well as diminished responsiveness to emotion are however consensually considered as central to apathy and are among the most frequent symptoms of bvFTD (81–83). Along with cognitive impairments, apathy is also fundamentally related to functional disability in terms of impaired instrumental activities of daily-living and it has a profound impact on patients' relatives (83–85). Immobility and reduced levels of daytime activity for example are seen in almost all patients with bvFTD and are a major source of caregiver distress (85). Along with cognitive impairments, apathy is also closely related to functional disability in terms of impaired instrumental activities of daily-living (86). Excessive TV watching, which may be observed more often in bvFTD than in AD may be perceived as a typical symptom of apathy (87), as well as a general reduction of interest toward pre-morbid activities. Importantly, apathy/inertia (i.e., “negative” symptoms) as well as disinhibited, agitated, or impulsive behaviors (i.e., “positive” symptoms) are no contradiction, but are often concomitantly present in patients with bvFTD and may be differentially triggered by certain situational cues (88). Also potentially related to overall levels of apathy, during anamnesis, patients may show a monotonous speech pattern that lacks modulation and prosody (89, 90). To assess specific symptoms in more detail, the Apathy Evaluation Scale [AES (91)] is available to clinicians.

#### Loss of Empathy or Sympathy

A diminution or lack of empathy is frequently observed in bvFTD in everyday social contexts and often has considerable negative consequences on caregivers (92). Lack of empathy relies on the interaction of several cognitive and sensorimotor processes (93, 94) and thus is a multifaceted symptom (95) that may be grossly defined as a deficit to share other's affective states (e.g., fear, sadness) leading to an absence of affective concern (and subsequently prosocial behaviors like comforting, helping or caring) for them. In its consensual definition, sympathy does not require one to share a specific affective state with someone else but would rather consist in an emotional reaction, such as sorrow or concern, to other's affective experiences (96). These symptoms could manifest as a diminution of projections into fictional situations such as in movies or books but more frequently, caregivers report a decreased spontaneous tendency to react to other's feelings (97). For example, patients could state their thoughts without considering the feelings of their colleagues or relatives (98). This detachment from friends and family go beyond what could be considered as social withdrawal—as observed in depression or apathy—because it also involves an “emotional blunting” about other's feelings, or, more generally, an overall lack of affective involvement. These deficits often lead to unmoderated, tactless, rough, and sometimes aggressive remarks from patients toward others, including their closest family, leading to what is often perceived as a very selfish and egocentric behavior (67). For instance, patients could be disinterested in spending time with their kids (99), they can be indifferent when their next-of-kin are hospitalized due to illness (98) or they may appear unimpressed when their partners cry during anamnesis. Empathy deficits often have a considerable impact on the family environment and are potentially dramatic for relatives and caregivers, especially as patients with bvFTD could overestimate their own empathic abilities (100). Overall, although empathy decrease can be observed in AD as well, it is substantially higher in bvFTD and plays a more important role in caregiver distress (33, 101). For a standardized clinical assessment of empathy deficits, the Interpersonal Reactivity Index [IRI, (102)], a caregiver questionnaire may be used.

#### Perserverative, Stereotyped, or Compulsive/ Ritualistic Behaviors

Patients with bvFTD frequently become rigid and inflexible regarding daily routines, which they want to preserve, and a substantial proportion develops unusual rituals or behaviors such as hoarding or collecting, similarly to what is observed in obsessive-compulsive disorders (103). Other anecdotally reported ritualistic behaviors are inflexible grooming or walking routines, counting and strict timekeeping as well as checking or sorting behaviors (14). However, in contrast to obsessive-compulsive disorder, these rituals are not due to compulsions in bvFTD as neither their expression nor their disruption are related to feelings of anxiety (104). Utterances during anamnesis may thus also appear stereotypic and repetitive and some patients present with additional verbal and/or simple repetitive motor tics (105). A proportion of patients develop a disinhibited drive to listen to music, and some may change their preferred music-style toward less complex music which they may consume in a preserverative manner (106). Creativity abilities have been shown to change during the disease, with patients exhibiting more naïve, stereotypic, and repetitive form of creation (107, 108).

#### Eating Behavior and Dietary Changes

Patients with bvFTD show significantly stronger changes in stereotypic/altered eating behaviors compared to AD patients (109). A marked hyperphagia (i.e., higher calory intake) has been found as quite specific for bvFTD when compared with other forms of neurodegenerative dementia syndromes, although patients with semantic dementia may also present with similar symptoms (110, 111). A craving for sweet food (“sweet tooth”) and a disinhibited, impulsive pattern of eating has been also frequently described as an early and specific sign of bvFTD, with sometimes patients stuffing aliments into their mouth and eating very quickly (binge eating). Patients with bvFTD may also be affected by hyperorality (e.g., Pica-syndrome, i.e., trying to eat inedible things or increased cigarette consumption) and various other dietary changes (112). While this section suggests that cognitive impairments are closely associated with changes in eating beahviors, it is important to highlight that elevated levels of leptin and insulin in bvFTD (as a direct consequence of hypothalamus atrophy) have also been linked to these symptoms (113, 114). The Appetite And Eating Habits Questionnaire [APEHQ (115)] may be used by clinicians to assess specific information on eating habits in a standardized way.

## Cognitive Dysfunctions in bvFTD

Although cognitive dysfunctions are numerically less weighted in the FTDC criteria for bvFTD, representing only 1 out of 6 criteria, the assessment of cognitive performance remains crucial for clinical diagnosis and patient management. Given that the previously described behavioral and psychological changes are often more visible in bvFTD, substantial cognitive impairments (that may account for some of the behavioral symptoms) may easily be overlooked. Recent studies have shown that the vast majority of patients with bvFTD already present with major cognitive dysfunctions in early disease stages when compared with normative data stratified for age and education (116, 117). Presymptomatic mutation carriers (with MAPT mutation) even present with specific (i.e., social cognition and memory) impairments respectively, 2 and 4 years before the diagnosis of bvFTD (118). Critically, commonly used multiple domain screening tests for dementia like the Mini Mental State Examination [MMSE (119)], the Montreal Cognitive Assessment [MoCA (120)] or even the more extensive Addenbrooke's Cognitive Examination [ACE-III (121)] may lack sensitivity to detect bvFTD in early stages and/or specificity to delineate the disease from other conditions, thus clearly warranting a more detailed neuropsychological assessment.

The neuropsychology item in the current criteria requires a cognitive profile of “executive/generation deficits with relative sparing of memory and visuospatial functions” which primarily aims at delineating the cognitive profile of patients with bvFTD from patients with AD (1). An array of recent studies however, revealed a large overlap between early bvFTD and other neurodegenerative diseases (including AD) regarding cognitive performances when using standard neuropsychological test batteries and when examining standard cognitive domains only (117, 122–125). We will thus now summarize the current evidence regarding the cognitive domains mentioned in the criteria (section Cognitive Dysfunctions in bvFTD) and subsequently review newer evidence regarding other cognitive domains (namely social cognition and praxis abilities) that are currently not represented in the criteria (section Promising Cognitive and Psychological Markers for bvFTD Beyond Current Diagnostic Criteria). We finally summarize evidence on impaired processing of interoceptive signals which may constitute a link between a range of cognitive and behavioral deficits in bvFTD.

### Executive Functions

Executive functions is an umbrella term for cognitive processes that rely on higher-order cognitive control mechanisms that are proposed to be mainly subserved by a large fronto-parietal network (126, 127). Although the proposed cognitive core deficits in bvFTD are executive dysfunctions, mixed results have been found when evaluating the (differential-) diagnostic properties of performance in neuropsychological tests for executive functions typically used in clinics (116). Executive dysfunction is a common symptom in a range of other neurodegenerative cognitive disorders including Parkinson's disease dementia (128), AD (129), dementia with Lewy-Bodies (130) as well as vascular cognitive impairment (131). When patients reach at least moderate disease severity stages, performances in tests for executive performance becomes strikingly similar across these diagnoses. But even in early stages, assessment of executive functions could not allow to always differentiate bvFTD from AD (116, 122, 125, 129, 132). In addition, executive impairments could be similarly present—or more severe—in a wide range of psychiatric disorders (133–135) and may thus provide only little help for differential diagnosis of bvFTD. Brief screening tools commonly used in clinics and specifically designed to rapidly assess a range of executive functions through a set of commonly employed single tasks [e.g., Frontal Assessment Battery (FAB) (136), INECO Frontal Screening (IFS) (137), FRONTIER Executive Screen (FES) (138)] have shown to effectively detect patients with bvFTD in the general population but have failed to consistently show sharp dissociations between different neurodegenerative dementia syndromes (139). Looking at standard single neuropsychological tasks for executive functions, performances of patients with bvFTD were frequently not unequivocally distinguishable from patients with other neurodegenerative dementia syndromes, including AD (117, 122, 125). This observation remains valid even with the use of more comprehensive evaluation relying on specific neuropsychological batteries such as the Executive Abilities: Measures and Instruments for Neurobehavioral Evaluation and Research (EXAMINER) (129) or the Delis-Kaplan Executive Function System (D-KEFS) (127, 140). When possible, the following part will address the different discriminatory ability of tasks related to classically defined executive domain in the differential diagnosis mainly of bvFTD and AD in early stages.

### Response Inhibition and Cognitive Flexibility

Disappointing results in terms of clear discriminative abilities have been shown for the Stroop tests (117, 141, 142), the Wisconsin-Card-Sorting test (143) and graphical sequences (142). Another commonly used test, the Trail-Making-Test part B (TMT-B) may even be more impaired in patients with AD compared to bvFTD (144) although bvFTD patients may be more insensitive to errors in this task (145). Environmental dependency symptoms such as imitation or utilization behavior have been described as more frequently observed in bvFTD than in AD (43, 72) but the variability of their assessment procedures and the probable multidimensionality of these symptoms (68) may have limited the investigations about their clinical relevancy and applicability. Even though saccade or anti-saccade abnormalities are sometimes reported as typical of bvFTD (46, 146), executive-related oculomotor function have been found to be impaired in AD as well (147), although they may be preserved in early disease stages (148). A range of studies have shown that the Hayling test may be a good candidate to more reliably distinguish patients between bvFTD and early AD on the level of individual cases (116, 149) but more research is needed to safely recommend the use of this test in this context test as some contradictory findings have also been reported (142, 150).

#### Abstract Reasoning

Verbal abstraction deficit as evaluated by categorization/similarities tasks is observed in bvFTD as opposed to AD, during the early stages of the disease (151, 152). These findings are in line with reported increased deficit in proverb interpretation in bvFTD compared to AD (153), although the semantic load of both tasks could also critically impact the performance due to polar temporal involvement (154). On the contrary, clock hand placement in the clock drawing test, hypothesized as involving the ability of abstracting the concept of time and its specific indication, have been shown to be altered preferentially in early AD as compared to bvFTD (155).

#### Initiation

Design/figural fluency tasks (e.g., 5-point test) are not differentially impaired in bvFTD compared to AD (144) although one study has found a higher number of qualitative repetition errors (156). Patients with bvFTD may score lower compared to AD patients in lexical fluency tasks (129, 157) and may also display a distinct ratio of semantic fluency vs. letter fluency with relatively less impairment in semantic fluency (158, 159).

#### Strategic Reasoning (Multitasking, Planning Abilities)

The Brixton test did not show any dissociation between bvFTD and AD (160, 161). This is also the case for the Tower of London test (162) although the use of qualitative information has been showed to have the potential to help the discrimination between both diseases (163). The Multiple Errands Test, the Zoo Map (from the Behavioral Assessment of the Dysexecutive Syndrome; BADS) and the Hotel task have all been found to be impaired in bvFTD (149, 164) but the lack of data in AD does not support their use in the context of clinical differential diagnosis. Cognitive estimation has been shown to be impaired in bvFTD as compared to amnestic MCI only (165).

#### Working Memory and Attentional Control

Auditory attention and verbal working memory deficits have been retrieved through digit span forward and digit span backward tasks respectively, (129, 143, 144) and were neither exclusively nor specifically impaired in bvFTD when compared with AD. Selective attention could be more impaired in bvFTD than in AD as well (141).

### Memory, Episodic Future Thinking, and Spatial/Topographical Navigation

Relative sparing of episodic memory remains a diagnostic feature in the neuropsychological criterion for bvFTD and has been historically heralded as one of the clinical gold standards to distinguish bvFTD from AD. However, this notion has been challenged by a study showing that bvFTD could present with severe amnesia, similarly to what is observed in AD (166). The critics that were made to this study (i.e., patients included only received clinical diagnoses and memory storage was only assessed through free recall testing) were addressed in an independent study with diagnoses supported by AD/non-AD biomarkers and memory assessment based on free and cued recall testing that showed similar results and proposed the existence of an amnestic variant of bvFTD following the observation of a bimodal distribution of patients (167). In the next years, several studies showed similar results and helped to further characterize the memory dysfunctions in bvFTD (168–170). A recent meta-analytic review confirmed a 37–62% overlap between bvFTD and AD in learning and recall test performance (171). Independent of the memory test used, the classic profile of bvFTD, i.e., a decreased spontaneous/free recall that can be normalized using recall cues could still be observed in about half of patients with bvFTD, however vast deficits in encoding, storage, and consolidation are present at a similar frequency. Likewise, recognition deficits have been observed in bvFTD (172, 173) further complicating differential diagnosis against AD. Interestingly, longitudinal studies have shown that early bvFTD could already present with severe amnesia (116, 132), in line with a neuropathological study of early FTD (174). While these two longitudinal studies showed a comparable rate of memory decline in bvFTD and AD, another one showed an even faster decline in bvFTD (175). Episodic future thinking, assumed to rely on the same neural mechanisms, has shown to be impaired in bvFTD (176) as well as the emotional enhancement of memory (177), in contrast to mild AD. Prospective memory is also impaired in bvFTD, similarly to AD, in both time-based (the ability to remember and execute an intended action at a future time) and event-based (when a specific event occurs) dimensions (178, 179). Both, recent and remote autobiographical memory were also shown to be impaired in bvFTD as well (180) but only in later stages of the disease (181). The only domain related to memory that seems to be robustly preserved in bvFTD as compared to AD is navigation ability. Topographical short-term memory has been found to be preserved in FTD (182), a finding which has been corroborated by recent studies showing that spatial orientation performance and particularly egocentric orientation (representation of spatial relationships in relation to separate objects) seems better preserved in early bvFTD and could thus allow an effective discrimination against early AD (183–185).

### Visuo-Spatial and Visuo-Construction Abilities

Drawing or copying of spatially complex (e.g., three-dimensional) figures and abstract forms (sometimes also referred to as constructional praxis) is a multifactorial process relying on a widespread bilateral neural network from temporo-occipital to parietal and lateral frontal areas (186, 187). From a cognitive stance, visual-perceptual abilities, planning abilities, and more general cognitive control processes are involved in figure-copy performance. Performance in tasks for visuoconstruction, such as drawing and figure copy abilities, has consistently found to be significantly more impaired in patients with AD as compared to bvFTD, and some studies suggest that visuoconstruction and visuoperception may be a specific and unique cognitive marker for preclinical AD (185, 188–191). In two samples (one of which included autopsy-proven FTD patients), patients with FTD had higher scores in the clock drawing test (192, 193). The domain of figure-copy abilities and visuoconstruction has further been shown to be particularly well-preserved in bvFTD over time in comparison to AD and other conditions (175). However, C9orf72 mutation carriers may be more likely to present with visuoconstructional deficits than non-carriers (61, 194). An in-depth assessment of visuospatial processing and visuoconstructional abilities e.g., using tests like the Rey-Osterrieth complex figure test (RCFT) is nevertheless highly recommended for the clinical differentiation between bvFTD and AD (195). Although some patients with bvFTD may still perform bad in some figure copy tasks, this is usually due to a deficit in perceptual organization and planning abilities and not due to a genuine visual-perceptual impairment (196, 197). It is thus recommended to qualitatively take into account the type of errors (e.g., planning deficits vs. spatial deficits) as spatial errors are particularly indicative of AD (198). There are several coding systems available to qualitatively study the types of errors in a visuoconstructive task like the Rey Complex Figure Test (199). Another specific qualitative error in figure-copy tasks that has previously been linked to executive dysfunction in patients with dementia is the phenomenon of “closing in,” in which the patient draws the copy very near to the model (200). It will be of particular interest whether qualitative assessment of this and other drawing errors may further enhance differential diagnostic efficiency for bvFTD.

### Summary of Cognitive Dysfunctions in bvFTD as Stated in the FTDC Criteria

Taken together, although the majority of studies have confirmed patients with bvFTD to show executive impairment, the proposed prototypical “profile” of pronounced executive dysfunction in bvFTD seems to be of low specificity and the cognitive dysfunctions displayed by patients with bvFTD are more heterogeneous than previously assumed. Although studies conducted in pre-diagnosed genetic bvFTD patients show that attention and executive functions could be early markers of MAPT or GRN mutations (118, 201, 202), they do not seem to precede other early cognitive impairments such as facial emotion recognition deficit or even memory storage impairments (118, 203). Furthermore, not all executive subdomains seem to be impaired in early bvFTD, questioning the notion of a general executive core deficit in bvFTD irrespective of disease stage (116). Small series of cases reports have indeed shown that in the earliest stages of bvFTD, executive functions may be normal (204). Overall, converging evidence rather suggests that only a subset of executive tasks that heavily rely on basic behavioral and motor inhibition abilities are robustly impaired in bvFTD, mostly irrespective of disease stage (116, 139, 142, 205).

## Promising Cognitive and Psychological Markers for bvFTD Beyond Current Diagnostic Criteria

In the following section we aim to review newer developments regarding the early detection of bvFTD mainly through cognitive domains currently not mentioned in the FTDC criteria and to give practical suggestions regarding their assessment in clinical routine.

### Social and Affective Cognition

Social cognition refers to a set of cognitive processes devoted to, or at least critically involved in, normal social interactions. This conceptual and somewhat arbitrary definition allows disentangling some mechanisms quite specific to the social dimension of cognition from others that may not be considered as specifically “social” despite their importance in interpersonal relationships such as language (206). Affective cognition may be defined as the mechanisms involved in emotional mediation of decision making and judgments, such as valence and reward processing, reinforcement, motivation etc. Both domains overlap widely as in one hand, mechanisms related to affective cognition often underlie social adjustment and in another, group, society, and culture could define or modulate the valence of behaviors. While this field of neurosciences is still emerging, studies that were focused on social and affective cognitive processing in bvFTD have flourished early in the 2000s and have contributed to develop our knowledge on the cognitive domain of social cognition besides helping the characterization of the disease (207, 208). In particular, these early studies showed that bvFTD patients could present a severe and early deficit in facial emotion recognition and theory of mind (or mentalizing), a cognitive ability allowing to infer other people's state of mind, such as what they want, think or feel. These pioneering works were followed by studies aiming to characterize the impairments in bvFTD in contrast to its most frequent differential diagnoses (209–214), to identify the neural correlates of these deficits (215, 216) and to finally enhance the accuracy and earliness of the clinical diagnosis (164, 212). In parallel, the exploration of social and affective cognition in bvFTD also enriched the computational modeling of cognition (217, 218), increased our understanding of the cognitive architecture (219, 220) and provided new theoretical models of social functioning (221). The prototypical social impairments observed in bvFTD have led many to explore social cognition functions in this disease, maybe more than in any other brain disease or neuroatypical functioning, except autism.

#### Theory of Mind, Mental State-Inference

An impairment in theory of mind abilities has been extensively described in bvFTD (222, 223). Deficits have been observed using different paradigms based on false-belief (224–226), detection of sarcasm (210, 227) or insincere communication (228), agency attribution (229), as well as emotional inference or attribution (209, 230, 231), emotional movement-based inference (232) and social faux pas detection and understanding (207, 209, 212, 213). While similar deficits may be observed in AD as well [e.g., (225, 229) for a review, see (233)], they seem to be variable and to depend on the severity of the disease. In contrast to AD, theory of mind deficits in bvFTD were only sparsely or not associated with other cognitive dysfunctions(209, 225, 232) and only little associations with other cognitive dimensions in bvFTD have been found when cluster-based or regression analyses specifically investing these relationships were employed (219, 220). Considering cases of selective impairment of theory of mind in bvFTD [e.g., (204)], and the vast evidence for theory of mind processing deficits across a wide range of different tasks (i.e., from simple first-order false belief task to more complex test requiring context and social norms processing such as in faux pas detection tasks), a primary deficit of theory of mind in bvFTD, somewhat independent of other cognitive dysfunctions becomes likely. In contrast to that, in AD, a secondary deficit (i.e., impacted by memory or executive dysfunctions) may be assumed (220, 223, 232, 234). However, the primary vs. secondary opposition needs to be refined and may lack of clinical relevancy for single case diagnoses until more reliable measures of theory of mind are available for clinical routine.

#### Emotion Recognition, Responding, and Expressiveness

Emotion recognition deficits in bvFTD have also been very well described since the earliest studies. Static (208, 235) as well as dynamic (236) facial expression of emotion have been particularly found to be under recognized in bvFTD despite increased eye fixation time being reported (237), with patients over-relying on external contextual information to decode the emotion (238). Emotion responding is also disturbed, with patients showing a diminished self-conscious emotional behavior (embarrassment and amusement) as well as a diminished associated physiological response, particularly toward negative emotional stimuli (239, 240). Similarly, blunted expressiveness in response to emotional stimuli, especially in low-intensity context and decreased autonomic responses such as skin conductance have been observed in bvFTD (241–244). Finally, recent investigations have showed that patients with bvFTD could have difficulties to imitate facial emotion expressions (245). In contrast, independent from the test considered, a systematic review recently conducted has shown that impairment in facial emotion recognition is not a consistent finding in AD and depends on disease severity (246–248).

#### Self-Related Representations and Agency

Self-related representations are impaired in bvFTD which is sometimes considered as a “prototypical disorder of the self” (249). Patients tend to overestimate their functioning in daily living activities, cognitive, emotional or motivational control, empathic, and social/interpersonal domains (100, 214, 250). As a consequence, they also underestimate their cognitive difficulties and have poor insight about their brain condition and its related management (214, 251). Patients could show inaccurate self-awareness of their current personality (252) and have diminished monitoring abilities and autonomic and emotional reactivity to errors in objective tasks (253, 254). There is also a diminution of the self-reference effect in bvFTD, known to increase performance in controls during memory processing (249).

#### Social Norms and Rules Processing Deficits

While social norms and rules processing deficits have never been assessed through objective testing in bvFTD unlike in other conditions [e.g., (255)], converging evidence suggest a global deficit in this domain. The differentiation within a culture between proper and improper behaviors that is made through morality mostly provided the most frequent context to assess this domain in the past 20 years. Findings from these studies reported no differences with controls on the evaluation (in terms of right or wrong) of conventional rules such as “how wrong is it if you keep money found on the ground” or in standard moral dilemma such as the Trolley Car Dilemma, in which patients have to adopt a utilitarian choice by deciding between causing the death of either one or five workmen (256). In addition, ratings of responsibility, blameworthiness and punishment were similar between bvFTD, AD and controls in a task involving moral transgressions in low (e.g., cheating for taxes) or high (e.g., murdering one own family) emotional context (257) in a deterministic context (i.e., without free will). However, differences between bvFTD and controls and AD were retrieved in the Footbridge Dilemma that is supposed to illicit empathic concern with a character of the story, as well as in evaluations of social and moral rules that have been considered as being grounded in mutuality and others' respect (256, 258, 259). Interestingly, patients were reported to verbalize less discomfort and also have reduced autonomic response during moral dilemma (256, 260) compared to controls. Lack of empathy, emotional blunting, and cognitive flexibility deficits have been considered as potential explanation for such deficits, as well as an impairment regarding the integration of social contextual information. More recently, the use of more sophisticated tasks to assess moral judgments showed that bvFTD over-rely on outcomes rather than intentions to consider attempted or accidental harms as permissible or not, therefore judging attempted harm as more permissible and accidental harm as less permissible than controls (261).

#### Reward Processing Deficit and Affective Decision Making

Only a few objective tasks have been used to assess reward processing in bvFTD but they showed that reward processing deficits are also very commonly observed in this disease. Patients could present a general deficit in reward valuation (262, 263) and thus, stimulus-reinforcement learning impairment (264). In patients in earlier stages of the disease who were not impaired in reinforcement learning, difficulties to suppress a previously rewarded behavior when it becomes punishing were observed (215), a lack of “reversal learning” that could prevent the quick adjustments that are needed on a daily basis in the social life. Although social rewards processing itself has been only rarely investigated in bvFTD, one study suggest that patients could be more indifferent to reward in comparison to AD and controls (265). Patients also showed a decreased sensitivity to negative stimuli (266) and delayed reward (267) as well as an absence of some natural decision-making bias such as the certainty effect, leading to a pathologically, albeit more rational, decision-making behavior (268). An innovative, laboratory-based, free-feeding study suggested that binge eating could be partially mediated by reward processing deficit as well, a result coherent with the fact that food is processed as a primary reward in the brain (269). In most of these different tasks, an AD group was included as a pathological control group and showed normal or subnormal performance in comparison to bvFTD. Reward processing deficits could thus be considered as an interesting cognitive marker of bvFTD but more reliable tasks are needed for clinical assessment.

Although the aforementioned tasks could be considered as assessing “decision-making” because they frequently involve binary choices between two items that are modulated by reward or punishment, the concept of decision making has mostly been retained to describe choices based on more complex information processing. In this context, apart from one exception (270), the Iowa Gambling Task has been employed, but revealed controversial results for the diagnosis of bvFTD. While early studies showed a good sensitivity (164) and specificity (209), others reported less clear results (213, 271), revealing a large intra-group variance in patients with bvFTD and AD as well as in controls that was dependent on levels of explicit knowledge individual participants had developed during the task [for a discussion, see (213)].

#### Symptomatic Behaviors of bvFTD and Social/Affective Cognition Impairments

While direct relationships between specific dysfunctions of social and affective cognition and abnormal behavior in bvFTD still have to be investigated in depth, social cognition assessment offers an objective evaluation of abilities that may drive many aspects of behavior. For example, a deficit in emotion recognition may prevent patients to recognize sadness, fear or anger in others' faces and to adapt their behavior accordingly. An impairment of theory of mind abilities would prevent patients to grasp others' mental states or feelings and thus to predict others' perspectives or actions, leading to behaviors that might appear egocentric or selfish. Similarly, a pathological theory of mind or empathy deficit could lead to commit abuses (223), or to be victim of one. The disintegration of social norms knowledge could also interfere with day-to-day adaptations in new social groups or contexts and may be associated with law violations in bvFTD (272). Alterations in reward processing may have an impact on the motivational aspects of what drives or regulates day-to-day behaviors, and could thus lead to apathy and lack of interest toward others, activities or things (83) and to a decrease of prosocial behavior (273). Likewise disinhibited eating patterns and binge eating has been associated with particular brain circuits involved in reward processing (269). Describing and delineating the different cognitive processes that drive or regulate behavior and that allow a smooth social life is thus among the biggest challenge in modern social and affective neurosciences [e.g., see the recent attempts to delineate apathy into distinct cognitive mechanisms (274, 275)].

#### Clinical Assessment of Social and Affective Cognition

The identification of social and affective cognition as a distinct cognitive domain [as in the current edition of the Diagnostic And Statistical Manual For Mental Disorders, DSM-5 (276)] allows researchers and clinicians to specifically target its mechanisms for research and clinical assessment. By contrast to behavioral scales or questionnaires such as the IRI, the Social Behavior Observer Checklist or the Social Norms Questionnaire (277), neuropsychological tests of social and affective cognition abilities offer an objective assessment, less impacted by inter-rater variability and by caregivers or clinicians' subjectivity. Thus, although the assessment of social and affective cognition is not mentioned in neuropsychological criteria of bvFTD, we recommend assessing at least one of the aforementioned functions when a social and/or affective cognitive impairment is suspected. Clinically suitable assessment tools differ widely in their length, reliability and norms availability. Clinical validity, i.e., an appropriate sensitivity and specificity for bvFTD is also a key criterion to guide the choice of a clinical tool. Although comparative validity studies are rare, some meta-analyses have been conducted to explore the ability of functions or tests to distinguish bvFTD from AD. For Facial emotion recognition, Ekman faces (278) or dynamic stimuli from the first part of The Awareness of Social Inference Test [TASIT (279)] are among the recommendable assessments in this perspective (280), while the later may not be available in languages other than English. A novel approach to assess emotion processing, which might be more sensitive to slight or subtle impairments is the rating of facial emotion intensity instead of labeling facial emotional expressions (281). For the assessment of theory of mind, Faux pas recognition tests and, to a lesser extent, sarcasm detection appear to be particularly useful to distinguish bvFTD from AD (233). However, particularly faux pas recognition tests are dependent on intact language comprehension, abstract reasoning and patient motivation, limiting their use to early stages of neurodegenerative cognitive disorders (231). Among validated batteries allowing the assessment of both functions (emotion recognition and theory of mind), the TASIT and the mini Social cognition and Emotional Assessment [mini-SEA; (212)] offer good clinical sensitivity and specificity. Beyond these tools mostly developed for or with patients in neurodegenerative diseases, the Wechsler Advanced Clinical Solutions Social Perception subtest (282), the Edinburgh Social Cognition Test (283) and the EMOTICOM [for Emotion, Motivation, Impulsivity, and Social Cognition (284)] could offer alternative multi-dimensional assessments of social cognitive functions with less language load, but their applicability in neurodegenerative syndromes has not yet been assessed.

### Apraxia

Still relatively poorly understood, apraxia is a multifactorial cognitive disorder affecting skilled movement, tool-use and/or gesturing on command despite intact task comprehension and basic sensorimotor functions (285). Impairments in praxis abilities can differentially involve imitation of limb gestures or face postures, the performance of communicative gestures (e.g., pantomiming the use of a common tool) or actual tool-use on command (286). Apraxic movements are qualitatively characterized by slow, insecure, and inaccurate movements which are performed in a halting and erroneous manner including frequent self-corrections (287). Historically, apraxia has been almost exclusively identified and studied in patients with left hemispheric stroke and comorbid aphasia (286, 288, 289). Nonetheless apraxia can also occur in the absence of aphasia, after e.g., right-brain lesions and also in a range of neurodegenerative disorders of which AD has been most extensively studied (290–295).

#### Apraxia and Impairment of Praxis Domains in Neurodegenerative Diseases

Despite explicit recommendations in consensus diagnostic guidelines for early neurodegenerative dementia syndromes (296) as well as its mentioning as a cognitive subdomain of the visual-perceptual abilities in the DSM-5 (276), the assessment of praxis disorders is currently widely neglected in clinical and neuropsychological diagnostic routine for patients with suspected dementia. Only recently, apraxia has gotten into the focus of clinical research on the early detection and differential diagnosis of neurodegenerative dementia syndromes such as bvFTD and AD (122, 297–300). Although results highly depend on the employed assessment methods and the tested praxis domains, an array of studies has now shown that patients with bvFTD show overall poorer performance in quantitative praxis tasks as well as clinical evaluations of praxis performance (297–301). Regarding the affected dimensions of praxis dysfunction, evidence suggests that performance in the domains imitation of meaningless hand or limb postures, pantomime of common object-use, and particularly imitation of face-postures are each significantly reduced in early stages of bvFTD compared to healthy age-matched controls (299, 302). These results are particularly intriguing as patients with bvFTD show no or only minor comorbid language symptoms in clear contrast to the prototypical apraxic and aphasic patients after left-hemispheric stroke. When compared to patients with AD, bvFTD patients present with a praxis profile of similar or less severe limb apraxia (i.e., imitation of meaningless gestures and pantomime of object-use) but relatively more pronounced buccofacial [or “orofacial,” sometimes used synonymously (303)] praxis deficits with a particular impairment regarding the imitation of face postures (122, 297, 299, 301, 302). Across different samples and using diverse apraxia tests such a relative “buccofacial apraxia profile” robustly showed high diagnostic accuracy for the diagnosis of bvFTD and also stood out among several established standard neurocognitive tests and domains (including standard memory and executive tasks) regarding diagnostic accuracy for the discrimination between AD and bvFTD (122, 301). Conversely, a praxis profile that is more indicative of AD (i.e., relatively more deficits in imitation of spatially complex, semantically meaningless limb gestures as compared to facial imitation) successfully predicted ß-amyloid levels (a core biomarker for AD) in the CSF of patients with a wide range of different clinical dementia syndromes (304). Although more research is needed, these results suggest that disease-specific praxis profiles may be related to underlying pathology beyond clinical presentations of neurodegenerative diseases.

#### Neural Substrates of Praxis Impairments

Regarding neural correlates of praxis impairments, large-scaled lesion studies in patients with stroke imply that limb praxis skills are subserved by densely interconnected but also segregated functional neuroanatomical networks involving parietal, temporal and frontal cortices mainly within the left hemisphere (305–308). More specifically, converging evidence point toward the involvement of at least two segregated neural “streams” for limb praxis [dual-stream model for action (309)]. A “dorsal stream” (leading from occipital visual areas via the parietal cortex into pre-motor areas) is suggested to be involved in gross visuospatial analysis of postures, online-sensorimotor control (e.g., important for the grasping of objects) as well as holding representations of learned skilled movements. For the latter function, neural correlates have been found primarily in the inferior parietal lobe (IPL) so that some authors subdivide this part of the dorsal stream into a “ventro-dorsal stream” (309). The ventral stream (leading from occipital visual areas via the temporo-parietal junction into the anterior temporal lobe) may be more involved with analysis of semantic aspects of gestures and movements including object identification and knowledge about the use and function of tools (307).

To our knowledge, only one study specifically investigated correlations between praxis performance and brain volume in patients with early stage bvFTD and AD so far (310). The authors found significant correlations between performance on the imitation of meaningless limb gestures and volumes of parietal cortices (primarily the IPL and the precuneus), mostly compatible with neural models of limb apraxia derived from patients with stroke. For object-pantomime, the results pointed toward a distinct involvement of the right hemisphere (correlation with middle right temporal gyrus and angular gyrus).

The neural correlates of deficits in buccofacial praxis abilities (as previously mentioned, a domain that is often specifically impaired in bvFTD) are however, mostly unknown regardless of whether investigating patients with stroke or neurodegenerative diseases. Anecdotal evidence however, points toward involvement of (medial) frontal areas and the frontal operculum in buccofacial praxis abilities and/or imitation of face postures (245, 311–313). Interestingly, buccofacial apraxia has also been reported in patients with MND (303).

#### Clinical Assessment of Praxis Impairments

Praxis dysfunction in bvFTD and other neurodegenerative dementia syndromes may well be tested with standardized assessment tools designed for stroke patients [e.g., Cologne Apraxia Screening (CAS) (314), Test of Upper Limp Apraxia (TULIA-AST) (315)]. However, these tests have not been validated in neurodegenerative dementia samples thus far and it has also not been addressed whether the praxis subdomains included in these tests are similarly relevant or important in neurodegenerative diseases. An exception with that regard is the Dementia Apraxia Test (DATE) (302), which has been constructed using a data-driven approach in order to maximize its utility for the early detection of patients with neurodegenerative dementia and particularly for differential diagnosis between AD and patients with FTD. The test is freely available, offers clinical cut-off scores for dementia (vs. healthy age-matched controls) and has been initially validated in patients with early AD, bvFTD and healthy elderly participants. The test has since also proven valuable for the differential diagnosis of language variants of FTD, showing evidence of disease-specific “apraxia profiles” (301). Data on praxis impairments in other early neurodegenerative patient groups (e.g., Parkinson's disease) as well as more extensive normative data for the DATE involving healthy controls from diverse cultural backgrounds is currently collected.

### Interoception and its Potential Links With Social Cognition, Apraxia, and Behavioral Symptoms of bvFTD

Interoception refers to the mechanism allowing us to perceive, infer, and predict our own physiological state through the integration of multimodal sensory input arising from the current state of the body (316). This sense of the body's internal states involves a large-scale brain system among which the insula, the thalamus, the anterior cingulate and the somatosensory cortices play a critical role (317). In bvFTD, interoceptive accuracy, and awareness have been shown to be decreased, notably in relation to fronto-insular gray matter and connectivity decrease (i.e., alterations in functional network connectivity). For example the so-called salience network, functionally connecting the main structures that were identified to be involved in interoceptive processing, has found to be attenuated in bvFTD (318, 319). Deficient processing of internal somatosensory signals in bvFTD has been shown across an array of different modalities including pain, temperature, and heartbeat perception, strengthening the hypothesis that the interoceptive function is a domain-general system supporting or at least strongly overlapping with emotion, motivation/reward processing and affective mental state inference (i.e., theory of mind abilities) (318, 320–324). Compared with healthy subjects, patients with bvFTD for example showed a lower autonomic response toward emotional stimuli (243, 244, 325), even when accuracy of detection was similar (326). Another recent study suggests that interoceptive impairment in different variants of FTD may be related to lower autonomic responses as well as to cognitive aspects of correctly analyzing body state representations (327). Because of the importance of emotion and theory of mind deficits in bvFTD as well as the increasing evidence pointing to interoception deficits and the associations of these deficits with insular damage, bvFTD may represent a prototype to investigate the relationship between these functions and could bring clinical data to understand the generative and predictive nature of the embodied mind.

With regard to links between apraxia, social and affective cognition, and interoception, preliminary data suggest that deficits in the imitation of facial postures may be correlated with facial emotion recognition performance and to a lesser degree also with caregiver reports of social-behavioral abnormalities in bvFTD (301). One reason for these associations and a potential common mechanism for both, facial affect recognition and face imitation may be that an accurate interpretation of internal somatosensory signals (and interoceptive changes) is required to (a) correctly decode and label a facial expression (or a body posture) and (b) to correctly imitate it (328). The broader idea of an “embodied cognition framework” for the perception of action stresses that interoceptive signals (e.g., muscle tonus, perception of spatial body postures) need to be correctly interpreted by the brain in order to then form accurate representations via internal motor simulations [e.g., motor imagery (329, 330)] and subsequently to accurately perform a gesture or posture (331–333). In other words, both accurate interoceptive signals and internal mental simulations (in terms of an accurate interpretation of these signals) may be a necessary and shared prerequisite for the ability to identify and correctly label face postures as well as to imitate them (334). Future research will eventually shed light on the precise associations between (facial) imitation abilities in praxis tasks, facial emotion recognition and processing of interoceptive signals as well as their biological and neural underpinnings in bvFTD and other neurodegenerative syndromes that share an early atrophy of brain regions crucial for interoceptive signal processing (e.g., semantic dementia).

## Conclusion, Challenges, and Outlook

Despite refined clinical diagnostic criteria, the early clinical diagnosis of bvFTD is still challenging and requires an in-depth assessment of clinical signs, behavioral and psychological symptoms as well as cognitive performance. Given the typically present anognosia in patients with bvFTD, caregivers are currently the main source of information for the evaluation of disease-typical behavioral and psychological changes. However, a standardized and focused neurocognitive assessment including memory, visuospatial abilities, social cognition, and praxis is crucial for an early differential diagnosis. Although executive dysfunction and preserved episodic memory are required to fulfill the “neuropsychology item” in the current criteria, we have highlighted that a range of studies have shown ambiguous results with more heterogeneous and complex cognitive performance deficits in patients with bvFTD. Neuropsychological assessments of a range of specific functions including aspects of social and affective cognition and praxis abilities have shown to potentially enhance the diagnostic accuracy but are not yet represented in the diagnostic criteria. It is however highly likely that behavioral symptoms e.g., a lack of empathy in bvFTD is mostly a behavioral expression of a social and affective cognitive deficit. Whether behavioral symptoms presented in e.g., psychiatric syndromes or in the phenocopy syndrome of bvFTD could also be captured by specific social and affective cognitive testing remains an open question and a challenge for the field.

In parallel of summarizing behavioral, psychological, and cognitive symptoms of bvFTD, we also presented and critically evaluated common methods of assessment for an early clinical diagnosis of this disease. We came to the conclusion that one of the major challenges of clinical neuropsychology in the coming years will be to fill the current gap of reliable and effective methods to assess cognitive alterations and access the behavioral and psychological symptoms as laid out in the criteria through performance-based testing. Neuropsychologists have to design novel tests that better fit the clinical practice and its requirements. Tools centered on diagnosis, evaluation, and follow-up are critically needed to replace some paper-pencil tests that are in use since several decades. Clinical neuropsychologists need to foster on new theoretical advances (e.g., psychological or neuroscientific models) and concrete (e.g., anatomical or biological) knowledge available in order to design novel ways of assessing cognitive functions and subsequently relate these to the typical behavioral symptoms. The development of new performance-based tasks is necessary to expand our evaluation to the currently over-looked domains of cognition in clinical routine, such as social cognition—a vast domains in itself, as well as reward processing, interoception, decision-making, and praxis abilities. However, in our opinion, there is also a vivid challenge to develop new ways of clinical assessment of the “classic,” well-known cognitive functions, such as memory, language, visuospatial processing, or executive functions. Despite the relevance they might have had at a certain time, it is surprising that tests such as the WCST (or its subsequent modified versions), originally published in 1948 (335) and developed to assess abstract reasoning and set-shifting in the normal population, are still used on a daily basis as a clinical test for prefrontal cortex function, despite knowledge about its numerous limits and shortcomings (336) including a lack of validity in non-western cultures (337). Similarly, the typical assessment of “episodic” verbal memory in clinical routine mostly relies on word-list that patients have to remember and recall, despite the lack of ecological value and autonoetic consciousness of this paradigm and the known confounding factor of semantic processing in cue-based recall tests (34). In particular, we believe that the social dimensions of cognitive key domains such as memory need to be taken into account in order to design more ecologically valid tests from which conclusions relevant for both clinical diagnosis and patients' activities of daily living may be drawn. In this perspective, individualized tests that use items specifically relevant to one patient [e.g., presenting faces from colleagues or friends to assess face familiarity (338)] are interesting, although certainly difficult to implement in clinical routine. Digital assessment technologies including computer-games or virtual reality are promising for clinical neuropsychology, however available apps frequently lack data on convergent validity with standard paper-pencil tests and normative data crucial for the interpretation of individual performance is rarely provided. Nevertheless, app-based cognitive research offers promising new opportunities by potentially increasing caregiver's and patient's motivation to participate in cognitive assessments, for example at home and thus with a maximum of comfort. Apps may also provide enormously large data-sets from which normative data and also very early abnormalities may be extracted using statistical “big-data” methods [for a successful example see a recent data analysis involving the app “Sea Hero Quest” (339)].

In the specific case of bvFTD, we believe that a major limitation to include some already well-described novel cognitive markers in the diagnostic criteria is the lack of availability of many current cognitive performance tests and behavioral scales. One of the potential reasons for this is an overall lack of research regarding cross-validation of the existing instruments as well as establishing adequate normative data and evidence of psychometric quality. These approaches require a lot of time and energy while being commonly disregarded in academia and research, as the former often leads to replications or negative-findings and the later will only be published in neuropsychology-specific, often national journals. This might be especially true for neuropsychological tools that are developed within a medical context (such as in neurodegeneration) as the difficulty to convince about the importance of psychometric studies and to conduct such studies in medicine may be higher than in the field of psychology. These shortcomings however, frequently impede a standardized interpretation of a patient's test result (by means of an evaluation of its abnormality) even when standardized performance tests or clinical scales are available for certain symptoms of bvFTD. Particularly, official translations into different languages are rarely provided or have usually not been independently validated in other cultures. In the case of diagnosing bvFTD, this is especially problematic as for example, perceived abnormality of social behaviors is highly culture-dependent. Such cultural variations may directly impact performance-tests as well as, for example, in the commonly used faux pas test: to mistake a young girl for a boy because she has short hair or a man for a waiter because he stands near the checkout in a restaurant may have been considered as rude and as a social faux pas in 1999 in the context of upper-class cantabrigian population in which the test has been originally developed, but may be perceived as simple mistakes without any negative social consequences for a lot of people 20 years later. Although cultural and socio-economical variability may appear as more important for the domain of social cognition, its influence may also be observed in a wide range of neuropsychological measures and “classic” cognitive domains [e.g., (340, 341)]. Finally, the vast majority of the presented standardized performance-tests and scales to differentiate between bvFTD and competing diagnoses (e.g., AD, affective disorders, other neurodegenerative diseases) have so far mainly been shown to function in usually small samples (with specific demographic and cultural characteristics), highlighting the importance of collaborative research and cross-cultural validation studies. Replications and cross-validation studies of tests and scales in larger and more diverse samples including extensive collection of data from healthy controls are needed in order to extrapolate from individual test results onto specific diagnostic decisions (e.g., Which cognitive performance profile or which set of behaviors are both pathological/abnormal and specific for an early diagnosis of bvFTD in a certain age-group, for a specific level of education, and in a certain culture?). In our view, this is currently one of the major challenges in the field of clinical neuropsychology but also a crucial prerequisite for the future establishment of valid and meaningful biomarkers for FTLD. In this perspective, the Social cognition and FTLD network (https://sites.google.com/view/soccogftld) has been created in order to share data on healthy controls and patients allowing large-scale clinical validity and cross-cultural studies—so far using the mini-SEA. If multiplicated, such initiatives may bring important data on both cognitive functions and the tests used to assess them and could later fasten the inclusion of specific cognitive dysfunction into the diagnostic criteria for neurodegenerative diseases.

An important future role of clinical neuropsychology will emerge with the evaluation and comparison of newly developed biomarkers by means of employing the complex assessment methods for behavioral, psychological symptoms as well as cognitive functions in patients with suspected bvFTD and competing diagnoses. Besides its role in cognitive rehabilitation, expertise in clinical neuropsychology will thus continue to play an important role in the detection and diagnosis of early bvFTD even and particularly in the light of emerging biomarkers for the disease.

## Author Contributions

All authors listed have made a substantial, direct and intellectual contribution to the work, and approved it for publication.

### Conflict of Interest Statement

MB would like to disclose that he is the main author of the mini-SEA. The remaining author declares that the research was conducted in the absence of any commercial or financial relationships that could be construed as a potential conflict of interest. The reviewer MI declared a past co-authorship with one of the authors MB to the handling editor.
